# LIGHT regulated gene expression in rheumatoid synovial fibroblasts

**DOI:** 10.1007/s11033-024-09311-0

**Published:** 2024-02-24

**Authors:** Koji Fukuda, Yasushi Miura, Toshihisa Maeda, Shinya Hayashi, Kenichi Kikuchi, Yoshinori Takashima, Tomoyuki Matsumoto, Ryosuke Kuroda

**Affiliations:** 1 Department of Orthopaedic Surgery, Kobe University Graduate School of Medicine, Kobe, Hyogo 650-0017 Japan; 2 Division of Orthopedic Science, Department of Rehabilitation Science, Kobe University Graduate School of Health Sciences, 7-10-2 Tomogaoka, Suma, Kobe, Hyogo 654-0142 Japan

**Keywords:** Rheumatoid arthritis, Fibroblast-like synoviocytes, LIGHT, Decoy receptor 3, Microarray assay, Gene expression profile

## Abstract

**Background:**

Synovial hyperplasia caused by rheumatoid arthritis (RA), an autoimmune inflammatory disease, leads to the destruction of the articular cartilage and bone. A member of the tumor necrosis factor superfamily, Lymphotoxin-related inducible ligand that competes for glycoprotein D binding to herpes virus entry mediator on T cells (LIGHT) has been shown to correlate with the pathogenesis of RA.

**Methods:**

We used cDNA microarray analysis to compare the expression of genes in rheumatoid fibroblast-like synoviocytes with and without LIGHT stimulation.

**Results:**

Significant changes in gene expression (*P*-values < 0.05 and fold change ≥ 2.0) were associated mainly with biological function categories of glycoprotein, glycosylation site as N-linked, plasma membrane part, integral to plasma membrane, intrinsic to plasma membrane, signal, plasma membrane, signal peptide, alternative splicing, and topological domain as extracellular.

**Conclusions:**

Our results indicate that LIGHT may regulate the expression in RA-FLS of genes which are important in the differentiation of several cell types and in cellular functions.

## Introduction

Rheumatoid arthritis (RA) is causes chronic inflammation in synovial tissues, resulting in synovial hyperplasia. Pannus forms, invades articular cartilage and bone, and causes joint damage. Characteristics of the hyperplastic synovial tissues of patients with RA have been reported, including the expression of oncogenes, apoptosis resistance, and somatic mutations [1, 2].

Lymphotoxin-related inducible ligand that competes for glycoprotein D binding to herpes virus entry mediator on T cells (LIGHT) or TNFSF14 is a member of the tumor necrosis factor (TNF) superfamily and is expressed by several cell types including T cells, monocytes, granulocytes, immature dendritic cells [3], synovial fibroblasts, macrophages [4], and endothelial cells [5]. LIGHT mediates T-cell activation and contributes to inflammation and apoptosis by binding HEVN, LTβR, and decoy receptor 3 (DcR3) [6]. Serum levels of LIGHT and LIGHT-positive lymphocytes are increased in patients with RA [4, 7] and LIGHT can contribute to synovial hyperplasia and joint destruction in patients with RA [7].

DcR3/TR6/M68/TNFRSF6b is a TNF receptor that binds three ligands: Fas ligand (FasL), LIGHT, and tumor necrosis factor-like ligand 1 A (TL1A) [8]. DcR3 overexpression may contribute to tumor development by reducing available FasL, LIGHT [6], and TL1A [9], thus reducing their cytotoxic and regulatory effects. Previously, we showed that DcR3, which is overexpressed in rheumatoid arthritis fibroblast-like synoviocytes (RA-FLS) and further induced by TNFα, protects cells from Fas-induced apoptosis [10]. Further, we reported that DcR3 binds to membrane-bound TL1A and may play a role in the pathogenesis of RA [11–13].

In previous studies, DcR3 [14], TL1A [15], and FasL [16] regulation of gene expression in RA-FLS, as demonstrated using cDNA microarrays, suggest that DcR3 signaling, as well as that of its two ligands, TL1A and FasL, is involved in RA pathogenesis. However, the role of LIGHT in RA pathogenesis is unclear.

In this study, we used cDNA microarray analysis to investigate LIGHT-regulated gene expression in RA-FLS. Results indicate molecules with significant roles in the LIGHT-HEVN/LTβR/DcR3 signaling pathway and RA pathogenesis.

## Methods

### Synovial fibroblasts

RA-FLS were obtained from four patients during total knee arthroplasty who fulfilled the criteria for RA of the American College of Rheumatology (ACR) in 1987 [17] or the ACR/European Alliance of Associations for Rheumatology (EULAR) in 2010 [18]. Patients were women aged 75.5 ± 10.3 years. Their levels of C-reactive protein were 1.15 ± 2.0 mg/dl (range, 0.10–4.17 mg/dl). Drug therapy for RA included conventional synthetic disease modifying-anti-rheumatic drugs (csDMARDs): 100 mg/day of bucillamine (two patients) or 10 mg/day of leflunomide (one patient). The fourth patient was treated with prednisolone (oral; 3 mg/day). Patients were excluded from the study if they had been administered biological disease-modifying anti-rheumatic drugs (bDMARDs) or Janus kinase inhibitors (JAKi).

Patients provided informed written consent to participate in this study in accordance with the Declaration of Helsinki and the study was approved by the Kobe University Graduate School of Health Sciences Ethics Committee (approval no. 308). Synovial tissues were cut up, cells were dissociated in Dulbecco’s modified Eagle’s medium (#D5796, DMEM; Merck, Darmstadt, Germany) containing 0.2% collagenase (Merck) for 2 h at 37 °C in 5% CO_2,_ and isolated cells were cultured in DMEM supplemented with 10% fetal bovine serum (#172,012, Merck) and 100 U/mL of penicillin (#45,397,387, Meiji Seika Pharma Co., Ltd., Tokyo, Japan) / streptomycin (#14,004,793, Meiji Seika Pharma Co., Ltd.) overnight at 37 °C in 5% CO2. Non-adherent cells were removed and adherent cells were provided fresh medium. Cells from passages 3 to 4 were used in this study [10].

### LIGHT-regulated gene expression

Individual RA-FLS cell lines (2 × 10^6^ cells/well of primary cultured RA-FLS) from patients 1–4 were incubated in either 1,000 ng/mL of recombinant human LIGHT protein (#664-LI/CF, R&D Systems, Minneapolis, MN) or OPTI-MEM medium (#31985-070, Thermo Fisher Scientific, Inc., Waltham, MA) for 12 h at 37 °C in 5% CO_2_. QIAshredder (#79,654, Qiagen, Hilden, Germany) and an RNeasy Mini kit (#74,104, Qiagen) were used to extract RNA from each cultured RA-FLS cell line, according to the manufacturer’s instructions.

Gene expression was detected using a microarray assay (Human Genome U133 Plus 2.0, GeneChip® 3’ Expression Array, Thermo Fisher Scientific), according to the manufacturer’s instructions.

### Statistical analysis

Data are represented as the mean ± standard deviation unless otherwise indicated. Microarray analysis were performed using Avadis 3.3 Prophetic software (Strand Life Sciences, Bangalore, India) [19]. *P*-values < 0.05 in a paired *t* test were considered statistically significant and a fold change ≥ 2.0 in expression was used to identify differentially expressed genes. Genes were ordered into hierarchical clusters by distance (Euclidean algorithm method) and linkage (complete algorithm method).

## Results

### Microarray analysis (gene expression profiling of LIGHT-stimulated RA-FLS)

The expression of over 53,500 genes was assessed using microarray analysis and results demonstrated that the expression of 1843 genes in RA-FLS were regulated by LIGHT. These genes were identified using the advanced search page of the UniGene database of the National Center for Biotechnology Information (https://www.ncbi.nlm.nih.gov/gene/advanced). Of the 1843 genes, 1042 differentially upregulated genes and 801 differentially downregulated genes (*P*-value < 0.05 and fold change ≥ 2.0) were identified in the LIGHT-stimulated group compared with the control group. Of the 1042 differentially upregulated genes, the UniGene database was able to annotate 814 genes. The top 20 upregulated genes of the 814 annotated genes are shown in Table 1. Similarly, 593 of the 801differentially downregulated genes were annotated. The top 20 downregulated genes of the 593 annotated genes are shown in Table 2.

**Table 1:** The 20 genes most upregulated by LIGHT. *P*-values were determined by a paired *t* test

Gene Symbol	*p*-value	Fold Change	Gene Title
BIRC3	0.000001	54.51	baculoviral IAP repeat containing 3
GABBR1///UBD	0.002026	26.14	gamma-aminobutyric acid (GABA) B receptor, 1///ubiquitin D
MEOX1	0.000001	22.65	mesenchyme homeobox 1
STAR	0.007237	13.29	steroidogenic acute regulatory protein
POU2AF1	0.000333	12.86	POU class 2 associating factor 1
C3orf80	0.000002	11.17	chromosome 3 open reading frame 80
RBP5	0.007259	10.64	retinol binding protein 5, cellular
FYB	0.014187	10.61	FYN binding protein
C11orf92	0.001560	9.71	chromosome 11 open reading frame 92
DAZL	0.000002	9.62	deleted in azoospermia-like
HSPD1	0.000215	9.53	heat shock 60 kDa protein 1 (chaperonin)
USP2	0.002784	9.40	ubiquitin specific peptidase 2
IL7R	0.007130	9.34	interleukin 7 receptor
OTTHUMG00000037055///U91328.2	0.000007	9.07	NULL///NULL
KY	0.000004	8.99	kyphoscoliosis peptidase
GRIA2	0.000307	8.77	glutamate receptor, ionotropic, AMPA 2
COL4A4	0.011861	8.70	collagen, type IV, alpha 4
GNRH1	0.004907	8.57	gonadotropin-releasing hormone 1 (luteinizing-releasing hormone)
TNFRSF9	0.000548	8.50	tumor necrosis factor receptor superfamily, member 9
AVPR1A	0.002350	8.04	arginine vasopressin receptor 1 A

**Table 2:** The 20 genes most downregulated by LIGHT. *P*-values were determined by a paired *t* test

Gene Symbol	*p*-value	Fold Change	Gene Title
INSC	0.019216	10.81	inscuteable homolog (Drosophila)
INIP	0.000073	10.33	INTS3 and NABP interacting protein
SNAP25	0.021064	9.86	synaptosomal-associated protein, 25 kDa
SEPT7P2	0.000051	8.74	septin 7 pseudogene 2
CRISP2	0.000278	8.45	cysteine-rich secretory protein 2
WNT16	0.009533	8.16	wingless-type MMTV integration site family, member 16
ARHGEF28	0.016078	7.85	Rho guanine nucleotide exchange factor (GEF) 28
LINC00327	0.000247	7.56	long intergenic non-protein coding RNA 327
OTTHUMG00000176187///RP11-202D1.2	0.001137	7.41	NULL///NULL
OTTHUMG00000181854///RP11-194N12.2	0.001024	7.38	NULL///NULL
FLJ32955	0.001947	7.12	uncharacterized protein FLJ32955
PAK2	0.000609	7.11	p21 protein (Cdc42/Rac)-activated kinase 2
AC007405.4///OTTHUMG00000154055	0.000729	7.03	NULL///NULL
LINC00628	0.012207	7.02	long intergenic non-protein coding RNA 628
OTTHUMG00000172555///RP11-8P11.2	0.004720	7.01	NULL///NULL
FGF7	0.000641	6.98	fibroblast growth factor 7
RAP1A	0.000555	6.84	RAP1A, member of RAS oncogene family
DIAPH1	0.002120	6.71	diaphanous-related formin 1
LINC00690	0.019198	6.68	long intergenic non-protein coding RNA 690
LMOD3	0.004996	6.40	leiomodin 3 (fetal)

### Functional annotation

The genes expressed differentially in response to LIGHT were classified by biological function (David Bioinformatics Database; https://david.ncifcrf.gov/). The 10 most significant categories of biological function were glycoprotein, glycosylation site as N-linked (GlcNAc…), plasma membrane part, integral to plasma membrane, intrinsic to plasma membrane, signal, plasma membrane, signal peptide, alternative splicing, and topological domain as extracellular (Table 3).

**Table 3:** The 10 most significant functional categories of the genes expressed by LIGHT exposure in RA-FLS

Category	Term	*P* value
SP_PIR_KEYWORDS	glycoprotein	0.000000001
UP_SEQ_FEATURE	glycosylation site: N-linked (GlcNAc…)	0.000000005
GOTERM_CC_FAT	GO:0044459 ~ plasma membrane part	0.000000005
GOTERM_CC_FAT	GO:0005887 ~ integral to plasma membrane	0.000000090
GOTERM_CC_FAT	GO:0031226 ~ intrinsic to plasma membrane	0.000000151
SP_PIR_KEYWORDS	signal	0.000000249
GOTERM_CC_FAT	GO:0005886 ~ plasma membrane	0.000000335
UP_SEQ_FEATURE	signal peptide	0.000000378
SP_PIR_KEYWORDS	alternative splicing	0.000000421
UP_SEQ_FEATURE	topological domain: Extracellular	0.000001101

## Discussion

Microarray analysis is a powerful method to investigate a wide variety of diseases, such as tumors [20], immunological diseases [21], and inflammatory diseases [22]. Previously, we used microarray analysis to characterize gene expression in DcR3 [14], TL1A [15], and FasL-stimulated [16] RA-FLS. Based on the gene expression profile of DcR3-stimulated RA-FLS, we examined in detail the expression and the function of IL-12B p40 [11], tryptophan hydroxylase 1 [13], and centrosomal protein 70 kDa [12] in DcR3-stimulated cells. In the gene expression profile of TL1A-stimulated RA-FLS, the expression of spectrin repeat-containing nuclear envelope 1, Fc receptor-like 2, PYD (pyrin domain)-containing 1, cell division cycle 45 homolog, signal transducer and activator of transcription 5B, and interferon regulatory factor 4 [15] were regulated by TL1A. In the gene expression profile of FasL-stimulated RA-FLS, FasL regulation of dual specificity phosphatase 6, epiregulin, interleukin 11, angiopoietin-like 7, protein inhibitor of activated STAT 2, and growth differentiation factor 5 [16] was identified.

In the present study, LIGHT regulation of baculoviral IAP repeat containing 3 (BIRC3), interleukin 7 receptor (IL7R), tumor necrosis factor receptor superfamily member 9 (TNFRSF9), synaptosomal-associated protein 25 kDa (SNAP25), and diaphanous-related formin 1 (DIAPH1) in RA-FLS was observed and classified into major functional clustering categories.

LIGHT upregulated the expression of BIRC3, which encodes cellular inhibitor of apoptosis protein 2 (cIAP2), to the greatest extent. cIAP2 is a component of nuclear factor kappaB and mitogen-activated protein kinase signaling pathways [23], and regulates cellular activities including differentiation, cytokine secretion, and cell death [24]. In RA, the therapeutic potential of cIAP2 is controversial. Previous reports have shown that cIAP2 deficiency is pro-inflammatory [25] and that antagonists against cIAP2 are anti-inflammatory [23]. In addition, antagonists against cIAP2 induce apoptosis in synovial fibroblasts [26].

IL7R expression was upregulated in the present study. IL7/IL7R signaling may play a role in various autoimmune or inflammatory diseases including multiple sclerosis, type 1 diabetes mellitus, RA, and ulcerative colitis. In patients with RA, IL7R expression is detected in monocytes, macrophages, synovial fibroblast cells, and endothelial cells [27, 28]. IL7/IL7R signaling may contribute to cell differentiation, such as that of T-cells, macrophages, endothelial cells, and osteoclasts, and to promote inflammation, angiogenesis, and joint destruction in RA pathogenesis [28].

Our results showed that TNFRSF9 expression was upregulated in response to LIGHT. TNFRSF9/CD137/4-1BB, an inducible costimulatory receptor, is expressed by monocytes and activated T and B cells. TNFRSF9 induces a signaling cascade in T cells that upregulates anti-apoptotic molecules, cytokine secretion, and enhanced effector function [29, 30]. TNFRSF9 signaling regulates the differentiation of Th17 cell through IL-6 expression in endothelial cells mediated by Akt and NF-kB pathways [31]. TNFRSF9 may play a role in autoimmune diseases [32] and immune response to infections [33], and may be a target molecule for cancer therapy [34]. Soluble TNFRSF9 is increased in RA patient sera [35], and the serum concentration of soluble TNFRSF9 and its ligand correlate with disease severity in RA [36].

SNAP25 was the third most downregulated gene observed in our analysis. SNAP25, a member of the soluble, N-ethylmaleimide-sensitive factor attachment protein receptor (SNARE) family, is important in neurotransmitter release and synaptic function [37]. In addition, SNAP25 is associated with the pathogenesis of cancers [38, 39] and some diseases affecting the nervous system and immune system in humans [40, 41].

DIAPH1 was downregulated in our analysis. DIAPH1 is a microtubule-binding protein that can nucleate actin filaments [42]. DIAPH1 is upregulated in laryngeal squamous cell carcinoma and may inhibit apoptosis in LSCC cells [43]. DIAPH1 knockdown promotes apoptosis in human glioblastoma cells [44].

In this study, we examined LIGHT regulation of gene expression in RA-FLS using a microarray assay. We recruited patients with a similar clinical background (never been treated with bDMARDs or JAKi and with severe knee joint destruction requiring total knee arthroplasty) to ensure sufficient sample homogeneity among the four samples.

The three ligands, TL1A, FasL, and LIGHT, bind the common decoy receptor, DcR3, and their activity is inhibited. Further study to understand the relationship between TL1A, FasL, and LIGHT regulation of gene expression should clarify the role of the TL1A/FasL/LIGHT/DcR3 signaling cascade in the pathogenesis of RA.

There are limitations to the present study. First, the study’s sample size is small. Second, microarray analysis demonstrates gene expression, but not mRNA or protein expression, which are required to confirm the expression of each gene in future studies.

## Conclusions

The present study examines the regulation of gene expression in RA-FLS by LIGHT. Our results indicate that the gene expression in RA-FLS regulated by LIGHT may be important in the activation and function of lymphocytes, proliferation, apoptosis, cytokine secretion, and intracellular signaling. The actions of LIGHT may be pleiotropic in the pathogenesis of RA, by not only improving but also aggravating RA. Our results may help clarify the pathogenesis of RA and identify of new targets for RA treatment.

## Data Availability

The microarray assay datasets are available in the Gene Expression Omnibus (GEO) repository of the National Center for Biotechnology Information. The GEO series accession number of the datasets is GSE197057 (https://www.ncbi.nlm.nih.gov/geo/query/acc.cgi?acc=GSE197057).

## Abbreviations

RA: Rheumatoid arthritis
LIGHT: Lymphotoxin-related inducible ligand that competes for glycoprotein D binding to herpes virus entry mediator on T cells
TNF: Tumor necrosis factor
DcR3: Decoy receptor 3
FasL: Fas ligand
TL1A: Tumor necrosis factor-like ligand 1 A
RA-FLS: Rheumatoid arthritis fibroblast-like synoviocytes
ACR: American College of Rheumatology
EULAR: European Alliance of Associations for Rheumatology
csDMARDs: Conventional synthetic disease modifying-anti-rheumatic drugs
bDMARDs: Biological disease-modifying anti-rheumatic drugs
JAKi: Janus kinase inhibitors
DMEM: Dulbecco’s modified Eagle’s medium
BIRC3: Baculoviral IAP repeat containing 3
IL7R: Interleukin 7 receptor
TNFRSF9: Tumor necrosis factor receptor superfamily member 9
SNAP25: Synaptosomal-associated protein 25 kDa
DIAPH1: Diaphanous-related formin 1
cIAP2: Cellular inhibitor of apoptosis protein 2
SNARE: Soluble N-ethylmaleimide-sensitive factor attachment protein receptor
